# Portable and Rapid Sequencing Device with Microbial Community-Guided Culture Strategies for Precious Field and Environmental Samples

**DOI:** 10.1128/mSystems.00748-21

**Published:** 2021-08-24

**Authors:** Ying-Ning Ho, Yu-Ling Chen, Ding-Yang Liu

**Affiliations:** a Institute of Marine Biology, College of Life Science, National Taiwan Ocean University, Keelung, Taiwan; b Center of Excellence for the Oceans, National Taiwan Ocean University, Keelung, Taiwan; c Department of Bioscience and Biotechnology, College of Life Science, National Taiwan Ocean University, Keelung, Taiwan

**Keywords:** cultivation, functional metagenomics, uncultured microbes, culture medium prediction, portable and rapid sequencing device, DNA sequencing, bioinformatics, environmental microbiology

## Abstract

Culturing unculturable bacteria is a classic microbiology challenge; to successfully culture unculturable bacteria, microbiologists work hard to create hundreds of culture conditions. To improve the success rate and efficiency of culturing a broad spectrum of environmental microbes, it is helpful to know more about the microbial community composition. Shortening the amount of time spent sequencing, analyzing sequencing data, and predicting suitable culture conditions seems to be a critical step for improving knowledge of microbes in environmental samples and expanding culture collections. In this commentary, we introduce potential strategies using rapid and portable sequencing devices to help scientists design and plan for specific microbial culture media on their way back to the laboratory. Rapid metagenomic sequencing approaches and real-time analysis make it possible to choose microbes we are interested in and discover novel microbes in environments for cultivation.

## COMMENTARY

The biggest misfortune is when you know treasure is by your side, but you cannot access it. More than a trillion (10^12^) microbial species have been predicted to inhabit the Earth; however, fewer than 20,000 bacterial and archaeal species have been cultured and validly described (1, 2). To culture the uncultured is a tough challenge for microbiologists but a sound investment with many rewards (3). For example, bacterial isolates are important for experimental examination to confirm inferences about their ecological roles and the cell biology of microorganisms from metagenomics analysis data. Fortunately, we are in the sequencing era: anything can be sequenced anytime and anywhere. Genomic sequencing approaches and accumulated bacterial information have engendered a “Cultural Renaissance” (4). Several innovative approaches have been developed for high-throughput or target cultivation such as cell sorting-based, membrane diffusion-based, and microfluidics-based cultivation (5). Some researchers, such as culturomics-based microbiologists, have tried to prepare multiple culture conditions for isolating novel and interesting microorganisms, while they face the precious samples the microbial information of which is unknown (6, 7). Identifying the unknown organism in samples at the time of collection would improve the success rate and efficiency of culturing.

## CULTURABLE OR UNCULTURABLE

“Unculturable is a frame of mind, not a state of microbiology,” Paul Carini wrote (4). Isolation efforts never stop. Many labs persistently try to bring novel microbes in to culture. Cultivars of 16S rRNA gene sequences and microbial assembly genome sequences have approximately doubled over the last decade, and ∼135,000 16S rRNA gene sequences and ∼346,000 whole-genome assembly sequences (about a third of all assembly sequences) were deposited into GenBank in 2020. However, the majority of cultivars are from human and human-associated environments (8). Most bacteria and archaea remain uncultivated, particularly in environmental habitats such as rocks, hydrothermal vents, seawater, marine sediment, aquatic systems, terrestrial subsurface, and soil (8). Many factors influence the culturability of environmental microbes: examples include physicochemical environmental conditions, substrates, growth conditions, symbiotic interdependencies, and low abundance and competition (5). How to resuscitate viable but nonculturable (VBNC) bacteria is an issue of concern (9). Furthermore, the knowledge gaps, personnel training, and infrastructure requirements can represent some barriers to isolation works. Fortunately, some cultivation methodology protocols and innovative methods have been developed for hunting a broad spectrum of novel microbes (5, 10), and many successful cases have been reported of culturing the uncultured (11, 12). These are probably called “the most wanted” microbes because they remain uncultured, such as SAR86, SAR202, SAR324, and “*Candidatus* Actinomarinidae” (4), but we think it is the last mile of the journey.

## KNOWN OR UNKNOWN

In the research and exploration of microbe cultivation, our knowledge of microbes remains between the known and the unknown. Yes, we could try to obtain the information of the total amount, bacteria size, and microbial diversity in environments, and even the genomic sequence of microbes including those culturable and unculturable by metagenomics, metatranscriptomes, and single-amplified genome sequencing (13). While we might expect that with knowledge of the microbial genome we can do anything, actually it is not immediately obvious to get the cultivated conditions and nutrient/substrate list that bacteria require for growth from genome sequences. The isolation of the abundant marine heterotroph *Pelagibacter* (SAR11) is a classic story. *Pelagibacter* has been cultured in natural seawater medium in 2002 (11). The whole genome of *Pelagibacter* has been sequenced and released in 2005 (14); however, it still takes more than 8 years to translate genome information into an artificial defined medium (15). How to translate bacterial genome information into a functional context is a key knowledge gap that needs to be solved to improve bacterial culturing. Some predicted genes in the genome sequence of novel microbes remain unknown and should be the investigated. We accept that “we don't know yet” and seek help from “nature.” Many researchers have used natural seawater medium (11), simulated natural environmental conditions (16), and even put microbes back into their original environments using membrane diffusion-based cultivation methods such as isolation chip (iChip) (17) to hunt unculturable microbes. However, scientists are never satisfied with questions remaining unanswered. Thanks to low-cost shotgun DNA sequencing and high-resolution mass spectrometry development, researchers can use the integrated omics approaches (metagenomics, metatranscriptomes, and metabolomics) to analyze and correlate with the microbial community (18). These approaches shed light on microbial metabolism *in situ* and provide key genes encoding enzymes that can predict the microbe’s preferences (19). Combining chemical profiling and its correlation with the microbial community might provide critical clues to design suitable culture media. These developed approaches and accumulated knowledge could be nutrients for culturing the “cultivation methods of unculturable microbes.”

## IMMEDIATE OR DELAYED

There are many unique and precious environmental samples such as samples from melting permafrost, tsunami, typhoon, and accidental pollution at a specific time point that may have only one chance for sampling (20). We need approaches that allow researchers to isolate as many microbes from these samples as soon as possible; otherwise, we risk losing the diversity. Some researchers want to isolate as many microbes as possible, prepare hundreds and thousands of culture medium plates for collecting microbes (6), and then use matrix-assisted laser desorption ionization–time of flight (MALDI-TOF) mass spectrometry—a fast and cost-effective method of taxonomic identification—to identify isolates and perform deduplication (21). In the same time, researchers can collect partial samples for sequencing (metagenomics and 16S rRNA target metagenomics) and analyzing microbial communities to compare the results of culture-dependent and independent methods.

**FIG 1:**
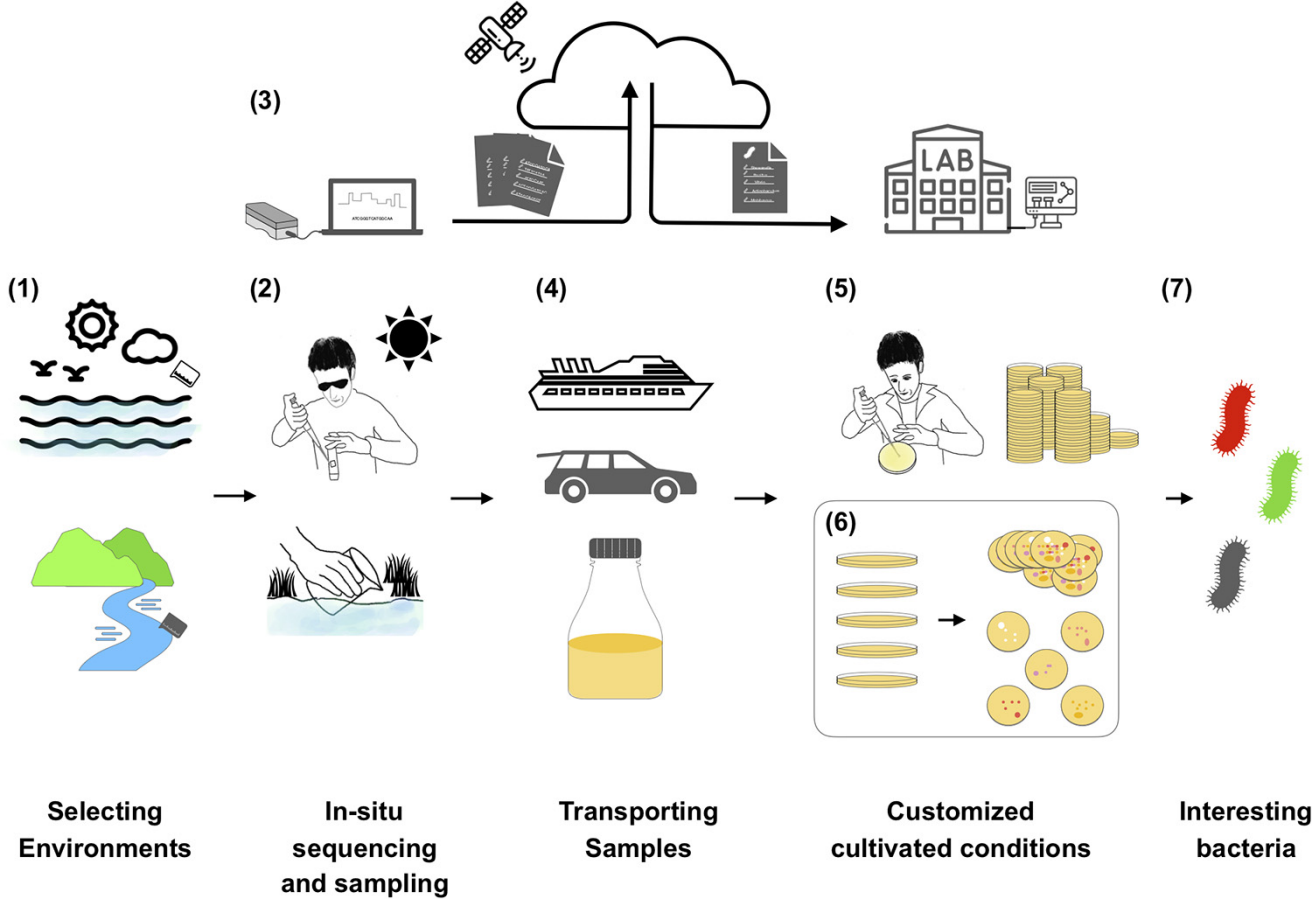
The workflow of microbial community-guided culture strategies. (1) We choose interesting environments for microbial collection. (2) We collect environmental samples and divide them into the sequencing and isolation parts. *In situ* DNA sequencing is launched within 30 to 70 min by Oxford Nanopore Technology. (3) The separated data (.fast5 or .fastq) are generated and immediately uploaded into the cloud space for microbial community analysis and culture condition prediction while the DNA is being sequenced. (4) We can take samples and the DNA sequencer back to our institute and lab. (5) In the same time, coworkers can obtain information about the microbial community and medium suggestions from the cloud computing space. (6) The customized medium plates are generated, and cultivated conditions are prepared for the incoming samples. (7) The interesting or target microbes are waiting for us.

Is it possible to obtain microbial genomic sequences and analyze data in real time to translate into cultivation information (culture medium, pH value, temperature, or oxygen required) when we return samples to the lab? Yes, it might be possible. The prototype of this system was developed in our lab by combining a portable and rapid sequencer, Oxford Nanopore Technology (ONT), and computer programs (unpublished data). Oxford Nanopore Technology (ONT) can be used in extreme fields—even off-Earth—for *in situ* DNA sequencing (22, 23). The equipment cost of Oxford Nanopore sequencing is suitable for a general laboratory. Therefore, we have built Flongle, MinON Mk1B, and Mk1C of the ONT system for metagenomics and 16S rRNA and internal transcribed spacer (ITS) amplicon sequencing. The maximum generated data are up to 34 Gb by the MinON and ∼1.3 Gb by the Flongle flow cell in our lab. Therefore, we use the advantages of ONT to construct our whole pipeline for microbe cultivation. The whole progress of the pipeline could be completed within 4.5 to 10 h including DNA extraction (30 to 50 min), DNA amplification (3 h, elective), DNA library (10 to 120 min), DNA sequencing and real-time data analysis of microbial community (3 to 4 h), and microbial community-guided culture medium prediction (5 to 10 min) (Fig. 1). The medium of a new organism could be predicted by existing culture medium-stain combinations (24). The web-based platform Known Media Database (KOMODO) has been built and proved by *in vitro* experiments (24). Based on this concept, resource, and database, we have optimized and built the system of microbial community-guided culture medium prediction for high-throughput data. We have used this system to predict rainwater, soil, marine sediment, and marine organism samples and get a high ratio of species-medium prediction (77.52 to 85.25%). However, these predicted species are not dominant microbes in environmental samples, especially in marine sediment (only 11.56%). Some limitations of the microbial community-guided culture system remain, such as missing information of minor microbes and the lack of culture information on microbes from new taxa or uncultivated microbes. The immediate sequencing of microbial DNA opens our eyes to designing suitable conditions that the target microbes need for growth.

## FURTHER APPLICATIONS

Rapid sequencing devices are used both in field-sample sequencing and in the fast and high-throughput screening of novel species. This system can combine the high-throughput culturing (HTC) methods (10). Sample inoculum is counted and diluted into a prepared medium-filled 96-well plate at 3 to 5 cells. After incubating and monitoring, we can take half the volume of bacterial culture for DNA extraction and full-length 16S rRNA amplification. This is not like pure culture sequencing, in which we separate individual colonies for Sanger sequencing. We can use direct sequencing for each well and then use the corrected full-length 16S rRNA sequence to distinguish the known bacteria from novel bacteria (25). One Flongle flow cell ($67.5 USD per cell) could sequence 16S rRNA for 60 to 96 wells, and the sequencing fee (∼$1 USD) is economical. We just chose the candidate wells and continued with further experiments. The information on a small bacterial population (each well) could also be used to discuss the symbiotic interdependencies.

This commentary is just the “starter” of rapid sequencing in “microbial research fermentation.” We believe that in the near future we will be able to achieve and culture previously uncultured species with the progress of technology and our continuous efforts.
